# Elevated levels of D-dimer in patients with COVID-19: prognosis value

**DOI:** 10.11604/pamj.supp.2020.35.2.24692

**Published:** 2020-07-07

**Authors:** Sara Oualim, Salma Abdeladim, Amal El Ouarradi, Ilham Bensahi, Sara Hafid, Abdelhamid Naitlho, Elarbi Bouaiti, Mohamed Sabry

**Affiliations:** 1 Department of cardiology, Cheick Khalifa International University Hospital, Mohammed VI University of Health Sciences (UM6SS), Casablanca, Morocco,; 2 Department of internal medecine, Cheick Khalifa International University Hospital, Mohammed VI University of Health Sciences (UM6SS), Casablanca, Morocco,; 3 Laboratory of Biostatistics, Clinical Research and Epidemiology, Faculty of Medicine and Pharmacy Mohammed V University, Rabat, Morocco.

**Keywords:** Coronavirus disease, D-dimer, prognosis, mortality

## Abstract

**Introduction:**

coronavirus disease is now a global pandemic due to rapid human-to-human transmission. It can cause mild to fatal respiratory, cardiovascular, and neurological diseases. We aimed to find out whether elevated D-dimer levels are a predictor of the bad progression of COVID-19 to help reducing the mortality.

**Methods:**

the data of COVID-19 patients from March 21, 2020 to April 24, 2020 were retrieved from the Cheick Khalifa Hospital database. We used the receiver operating characteristic (ROC) curve to get the optimum cutoff value of D-dimer levels on admission and after 5 days. We used these cutoffs to divide patients into two groups and compare the in-hospital mortality between them to assess the prognosis value of D-dimer levels.

**Results:**

the data of COVID-19 patients from March 21, 2020 to April 24, 2020 were retrieved from the Cheick Khalifa Hospital database. We used the receiver operating characteristic (ROC) curve to get the optimum cutoff value of D-dimer levels on admission and after 5 days. We used these cutoffs to divide patients into two groups and compare the in-hospital mortality between them to assess the prognosis value of D-dimer levels. 89 patients were included in this study, of whom 79 were discharged and 10 died in hospital. The optimum cutoff value to predict mortality in patient using D-dimer levels on admission was 668 ng/ml (sensitivity 90%, specificity 63.3%, Areas under the ROC curve 0,775). As for D-dimer levels on day 5, it was 1360 ng/ml (sensitivity 100%, specificity 88,6%, Areas under the ROC curve 0.946). The group with D-dimer levels on day 5 > 1360 ng/ml (19 patients) had a worst evolution and a higher incidence of mortality compared to the group with D-dimer < 1360 ng/ml (69 patients) (10/19 vs 0/69, P = 0,0002).

**Conclusion:**

D-dimer greater than 1360 ng/ml on day 5 could help clinicians identify patients with poor prognosis at an early stage of COVID-19.

## Introduction

Since December 2019, a cluster of pneumonia has attacked all human beings [1]. The pathogen was designated as SARS-CoV-2 by the International Committee on Taxonomy of Viruses, and this pneumonia was named as Coronavirus Disease 2019 (COVID-19). This new infective outbreak has spread quickly all around the world [2]. The challenge was to find effective predictors of COVID-19 critical disease and death in order to identify critical patients early. Elevation of D-dimer indicated an hypercoagulable state in patient with Covid-19 and reflects activation of coagulation and fibrinolysis [3]. Recent literature data show that D-dimer values are frequently enhanced in non-survivor patients with COVID-19 [4,5]. In this article, we analyzed the prognosis value for D-dimer on admission and after 5 days. This would help us predict the progression of the disease and adjust the treatment plan to reduce the risk of death.

## Methods

**Study design and participants:** this retrospective study was conducted in Cheick Khalifa International University Hospital, Mohammed VI (Casablanca, Morocco). All adult patients (≥18 years old) who were diagnosed with COVID-19 according to WHO interim guidance, and confirmed by RNA detection of the SARS-CoV-2 in onsite clinical Laboratory, were screened. Our study enrolled all adult inpatients who were hospitalized for COVID-19 with D-dimer level on admission, five days later and a definite outcome (dead or discharged), between March 21, 2020 and April 24, 2020. Therefore, we excluded patients with incomplete data, leaving 89 includes of the 149 patients originally reported on. The study was approved by the Research Ethics Commission of Cheikh Khalifa Hospital and the requirement for informed consent was waived by the Ethics Commission.

Data collection: epidemiological, clinical, laboratory, and outcome data were extracted from electronic medical records using a standardized data collection form. All data were checked by two physicians (SA and SH) and a third researcher (SO) adjudicated any difference in interpretation between the two primary reviewers.

**Laboratory assay and intervention:** throat-swab specimens were obtained for SARS-CoV-2 PCR examination. The criteria for discharge were absence of fever for at least 3 days, clinical remission of respiratory symptoms, improvement in both lungs in chest CT, and two throat-swab samples negative for SARS-CoV-2 RNA obtained 24 h apart. Blood samples were collected within 24 hours after admission to perform routine laboratory tests, such as blood count, coagulation profile, renal and liver function, creatine kinase, lactate dehydrogenase, D-dimer and myocardial enzymes. A second blood sample was collected the fifth day to control D-dimer level. D-dimer was determined on VIDAS D-Dimer Exclusion II (DEX2) by utilizing Enzyme Linked Florescent Assay (ELFA). The laboratory reference range was 0-500 ng/ml. The D-dimer result was expressed in ng/ml FEU (Fibrinogen Equivalent Unit). All measurements were done within 2 hours after blood sampling.

**Statistical analysis:** continuous and categorical variables were presented respectively as median (IQR) and n (%). We used the Mann-Whitney U test and χ2 test to compare differences between the two groups. Event frequencies were compared with chi-square test. Mortality discrimination for D-Dimer levels was calculated using the receiver operating characteristic (ROC) curve. The optimal D-dimer cutoff point and C-statistic of routine laboratory tests were evaluated. We used Spearman correlation to assess correlation between D-dimer levels on admission, on day 5 and the length of hospital stay. We divided the subjects into two groups according to D-dimer levels on admission and on day 5 and we compared the in-hospital mortality between the two groups to assess the prognosis value of D-dimer levels using Kaplan-Meier survival analysis. A value of p<0.05 was accepted as statistically significant. The statistical software package MedCalc Statistical Software (version 16.2, Ostend, Belgium) were used for data analysis.

## Results

**Baseline characteristics:** 149 adult patients were hospitalized in Cheick Khalifa Hospital with COVID-19 between March 21, 2020 and April 24, 2020. After excluding 60 patients without available key information in their medical records, we included 89 inpatients in the final analysis. Ten patients died during hospitalization and 79 were discharged. The median age of the 89 patients was 48 years (IQR, 28- 68 years), ranging from 18 years to 84 years, and most patients were male (Table 1). Comorbidities were present in nearly 44.9% of patients, with hypertension being the most common comorbidity, followed by diabetes and coronary heart disease. The most common symptoms on admission were fever, cough, headaches, followed by dyspnea and fatigue. Table 1 contains the basic characteristics of the patients. Among routine laboratory tests, D-dimer on Day 5 has the highest C-index to predict in-hospital mortality in COVID-19 patients. Besides, the C-indices indicates C-reaction protein and creatinine, are also strong predictors for these patients (Table 2).

**Table 1:** baseline characteristics of 89 patients with COVID-19

Variable	Total n = 89	D-dimer on admission < 668 n = 55	D-dimer on admission > 668 n = 34	p-value	D-dimer on day 5< 1360 n = 69	D-dimer on day 5> 1360 n = 20	p- value
**Age-yr. ( IQR)**	48 (28-68)	45 ( 25- 65)	52 ( 33- 71)	0.40	43 ( 23- 63)	57 (26- 88)	0.09
**Age > 65 yr-n(%)**	26 (29.2)	13 (26)	13 (33.3)	0.45	17 ( 24.6)	9 ( 45)	0.078
**Female- n (%)**	39 (43.8)	20 (40)	19 ( 48.7)	0.41	30 (43.5)	9 (45)	0.9
**Underlying conditions-n (%)**	40 (44.9%)	21 (20)	19 (55)	1.1	25 (36)	15 (75)	1.4
**Hypertension-n (%)**	22 (25)	11 (22)	11 (28.9)	0.45	15 (22)	7 (35)	0.24
**Diabetes-n (%)**	12 (13.6)	7 (14)	5 (13.2)	0.90	6 (8.8)	6 (30)	0.015
**Coronary heart disease-n (%)**	5 (5.7)	2 (5.3)	3 (6)	0.88	3 (4.4)	2 (10)	0.34
**Chronic kidney disease-n (%)**	1 (1.1)	1 (2.6)	0 (0)	1.33	1 (1.5)	0 ( 0)	0.58
**Symptoms**							
**Fever-n (%)**	52 (58.4)	22 (56.4)	30 (60)	0.73	40 (58)	12 (60)	0.87
**Cough-n (%)**	51 (57)	20 (51.3)	31(62)	0.31	42 (60.9)	9 (45)	0.20
**Headaches-n (%)**	26 (29.2)	11 (28.2)	15 (30)	0.85	21 (30.4)	5 (25)	0.63
**Dyspnea-n (%)**	24 (27.3)	8 (21.1)	16 (32)	0.25	17 (25)	7 (35)	0.37
**Fatigue-n (%)**	18 (20.2)	9 (23.1)	9 (18)	0,55	14 ( 20.3)	4 ( 20)	0.97
**Routine tests on admission**							
**WBC- 10^3^/mm^3^ (IQR)**	6.5 (4.09, 8.6)	6.71 (4.56, 8.53)	6.24 (4.2, 8.26)	0.16	6.69 (4.8, 8.5)	6.8 (4.8, 8.8)	0.760
**Hemoglobin-g/dl (IQR)**	14 (12.7, 15.3)	14.1 (12.6, 15.5)	13.8 (12.5, 15.07)	0.46	13.5 (12.7, 14.3)	14.2 (13.5, 14.9)	0.41
**Platelet-10^3^/mm^3^ (IQR)**	241 (164, 318)	249 (174, 328)	232 (153, 311)	0.07	229 (120, 338)	250 (177, 323)	0.6
**CRP- mg/L (IQR)**	43 (3, 107)	32 (2, 62)	58 (3, 113)	0.024	36 (4, 68)	67 (5, 129)	0.002
Creatinine- mg/L (IQR)	9.3 (3.3, 15.3)	8.77 (4.47, 13.07)	11.18 (1.9, 20.3)	0.001	10 (6.5, 14.5)	15.6 (2, 28)	<0.001
**D-dimer ng/ml on Day 5**	1204 (1, 3300)	555 (10, 1767)	2011 (13, 4627)	<0.001	/	/	/
**Severe COVID-19 - n (%)**	37	12	25	<0.001	19	18	<0.0001
**Hospital stay days**	12.3 (7.3, 17.3)	10.4 (7, 13.8)	14.8 ( 9, 20.6)	0.008	11 (7, 15)	17 ( 10, 25)	0.005
**Non survivors-n (%)**	10 (11.2)	1 (0.018)	9 ( 26.4)	<0.001	0	10	< 0.001

Data are mean±SD, median (IQR), n (%). p values were calculated by t test, Mann-Whitney U test, χ^2^ test, or Fisher’s exact test, as appropriate. IQR: inter-quartile range; CRP: C-reaction protein.

**Table 2:** C-statistic of routine tests to predict mortality in patients with COVID-19

Routine laboratory tests	C-index	95% CI
**D-dimer on admission**	0.775	0.674- 0.857
**D-dimer on Day 5**	0.946	0.876- 0.982
**C-reactive protein**	0.910	0.831- 0.960
**Platelet**	0.631	0.522- 0.731
**Creatinine**	0.824	0.729- 0.897
**White blood cells**	0.547	0.438-0.653

CI: Confidential interval

**Figure 1:**
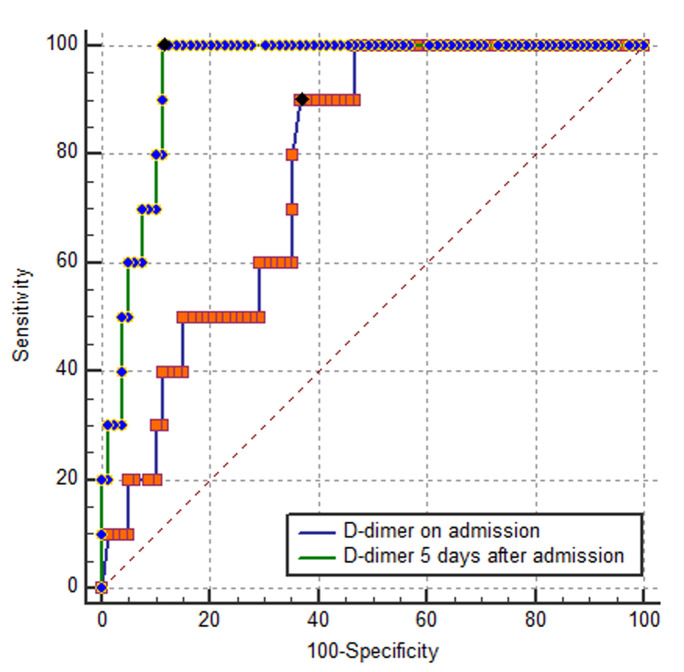
ROC curve showing validity of D-Dimer values in predicting mortality for COVID-19 patients.

**Figure 2:**
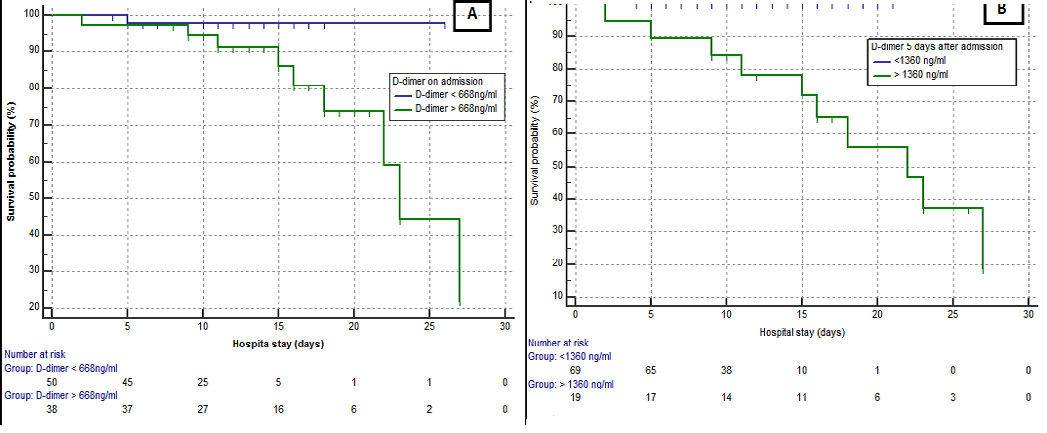
Kaplan –Meier survival curves: (A) for D-dimer levels on admission (p = 0.11); (B) for D-dimer levels on day 5 (P = 0.0002)

**Figure 3:**
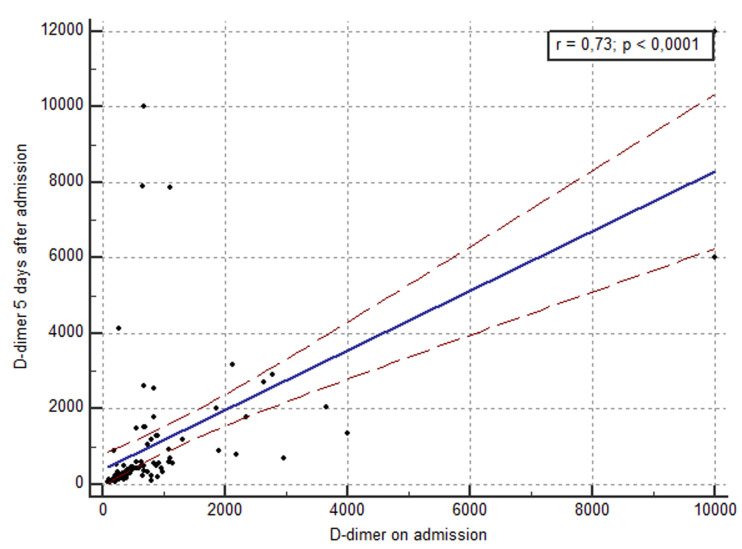
scatter graph showing correlation between D-dimer levels on admission and 5 days after admission (r = -0.73; p <0.001)

**High D-dimer levels to predict mortality:** best cutoff points to predicting mortality in patient with COVID-19 using D-dimer levels on admission was 668 ng/ml (sensitivity 90%, specificity 63.3%%, area under ROC curve 0.775). As for D-dimer levels on day 5, it was 1360 ng/ml (sensitivity 100%, specificity 88,6%, area under ROC curve 0.964). There were statistical differences among D-dimer levels on admission and on day 5 in terms of area under the ROC curve (p = 0.002) (Figure 1). According to the cutoff value, 55 patients D-dimer levels on admission were less than 668 ng/ml, 34 patients had D-dimer levels over 668 ng/ml. 69 patients had D-dimer levels on Day 5 less than 1360 ng/ml and 20 patients had D-dimer levels overs 1360 ng/ml. A total of 10 death events occurred during hospitalization, nine of them were observed among patients with D-dimer levels ≥668 ng/ml on admission. Only one event occurred in patients with D-dimer levels (<668 ng/ml) on admission (9/34 vs. 1/55). As for D-dimer levels on day 5, we observed zero death in the group with D-dimer < 1360 ng/ml (10/20 vs 0/69). Kaplan-Meier Survival Curves for D-dimer, revealed that the group with D-dimer levels on day 5 > 1360 ng/ml had a worst evolution and a higher incidence of mortality compared to the first groups with D-dimer levels on day 5 < 1360 ng/ml (10/19 vs 0/69, P = 0,0002) (Figure 2). In addition, D -dimer levels on day 5 are significantly correlated with the D-dimer levels on admission (r=0.73, p<10-4) but D-dimer on admission and on day 5 failed to show any significant correlation with length of hospital stay (r=0.28 and r=0.29 respectively) (Figure 3).

## Discussion

This study showed that D-dimer on day 5 greater than 1360 ng/ml was associated with higher odds of in-hospital death. it was more commonly seen in severe COVID-19 illness as well. It confirmed that increased D-dimer levels on day 5 was a mortality predictor. The study established a cutoff value as well, to identify at an early stage the patients with COVID-19 who might suffer and have a bad prognosis. D-dimer dynamics can reflect the severity and their increased levels are associated with adverse outcomes among patients with community-acquired pneumonia [6]. Recent literature data show that D-dimer values are frequently enhanced in patients with COVID-19 and that it is even higher in patients with severe COVID- 19 than in those with milder forms. Accordingly, elevated D-dimer was detected in 36% of patients in a descriptive study of 99 COVID-19 cases in Wuhan, China [7]. Huang et al reported clinical and laboratory data of 41 patients hospitalized with confirmed COVID-19 and observed that D-dimer values were fivefold higher in those with severe disease (median: 2.4mg/L; IQR: 0.6-14.4mg/L) than in those without (median: 0.5 ng/mL; IQR: 0.3-0.8mg/L; p¼ 0.004) [6]. Tang N et al found that non-survivors had a significantly higher D-dimer than that of survivors [4]. Wang et al included 138 patients hospitalized for COVID-19, D-dimer values were nearly 2.5-fold higher in patients with severe disease than in those without [8]. Zhou et al studied 191 patients with COVID-19 and found that D-dimer values were nearly ninefold higher in patients who died than in those who survived [9]. None of this studies provide well evaluated cutoff for D-dimer.

In a multicenter retrospective study during the first two months of the epidemic in China, 260 out of 560 patients (46.4%) with laboratory confirmed COVID-19 infection had elevated D-dimer (≥ 500 ng/ml). The cut-off (i.e., 500 ng/m) was locally defined. The risk of having D-dimer values above 500 ng/ml was more frequent in patients with severe disease than in those without [10]. Zhang et al extracted data on 343 patients enrolled in Wuhan with COVID-19. The cutoff value of 2000 ng/ml (fourfold increase) for D-dimer was established by ROC curve. D-dimer levels ≥2000 ng/ml had a higher incidence of mortality when comparing to those who with D-dimer levels < 2000 ng/ml [11]. In current study, a clear cutoff value (668 ng/ml on admission and 1360 ng/ml on day 5) for D-dimer was well established by ROC curve. We found that of the 10 non-survivors with D-dimers over 1360 ng/ml, three patients had no severity symptoms on admission. Nonetheless, what clearly emerges from this results is that even in the absence of other severity symptoms, patients who have high D-dimer levels over 1360ng/ml on day five should be closely monitored. This highlighted the fact that D-dimer measurement may be associated with evolution toward worse clinical picture. In addition, we found a significant correlation between D-dimer levels on day 5 and D-dimer levels on admission. This means that serial measurement of D-dimer would help recognizing at an early stage, the COVID-19 patients who might have a poor prognosis.

In fact, elevation of D-dimer indicated a hypercoagulable state in patient with COVID-19 [12]. It can be attributed to many reasons. SARS-CoV-2 can cause direct myocardial injury. It infects cardiomyocytes by identifying ACE2 receptor [13]. Indirect injury may be caused by inflammatory storm. Various inflammatory factors produced by this storm might induce the dysfunction of endothelial cells, resulting in excess thrombin generation [14]. Current studies have shown that up to 20% of covid-19 patients have abnormal coagulation function [10]. The hypercoagulability of blood will increase the risk of thrombosis and embolization of the visceral causing ischemia and hypoxia, which leads to the progression of the disease to critical disease or death. In fact, critical covid-19 patient´s dissection showed occlusion and micro-thrombosis formation in pulmonary small vessels [15]. Tang et al also recently reported that the vast majority of COVID-19 patients who died during hospital stay fulfilled the criteria for diagnosing sepsis-induced coagulopathy or disseminated intravascular coagulation (71.6 vs. 0.6% in survivors) [4]. To enhances the precision of scores for the identification of high venous thromboembolism (VTE) risk patients, a modified IMPROVE-VTE Risk Assessment Models which includes the D-dimers levels together with other clinical predictors of VTE was made [16-18]. This study has some limitations. First, interpretation of our findings might be limited by the sample size and the fact that it was a retrospective study. However, we believe we had enough information to find significant differences between the groups in mortality. Second, we excluded 60 patients for unavailable data (absence of D-dimer on admission). Some patients were even transferred late in their illness. This might have contributed to change the results. Third, by excluding patients still in hospital as of Apr 24, 2020, and thus relatively more severe disease at an earlier stage, the case fatality ratio in our study cannot reflect the true mortality of COVID-19.

## Conclusion

D-dimer elevations may be commonplace in patients with severe forms of COVID-19 as in other severe infections disease. Their careful evaluation and monitoring could effectively predict in-hospital mortality in patients with COVID-19 at an early stage. This would assist clinicians in formulating a tailored treatment approach and promptly provide intensive care to those who are in greater need.

**What is known about this topic**

- Measuring D-dimer had been recommended for COVID-19 patients;
- The management of COVID-19 patients might be improved by finding early mortality markers.

**What this study adds**

- The optimal cutoff for D-dimer to predict in-hospital mortality for COVID-19;
- The prognosis value of D-dimer measurements on admission and after 5 days.
